# Decision regret after partial nephrectomy for benign small renal masses: The importance of preoperative counselling

**DOI:** 10.1002/bco2.70289

**Published:** 2026-10-16

**Authors:** Sarika Nalagatla, Aqua Asif, Bhargab Ghoshal, Maria Yacoub, Samuel Davis, Maria Bahhadi‐Harbo, Abhishek Reekhaye, Manar Malki, Muddassar Hussain, Neil Barber

**Affiliations:** ^1^ Department of Urology Frimley Park Hospital, Frimley Health NHS Foundation Trust Surrey UK; ^2^ Division of Surgery and Interventional Science University College London London UK; ^3^ University College London Medical School London UK; ^4^ Histopathology Department Frimley Park Hospital, Frimley Health NHS Foundation Trust Surrey UK

**Keywords:** benign renal tumour, decision regret, partial nephrectomy, patient‐reported outcomes, preoperative counselling, renal mass biopsy, shared decision‐making, small renal mass

## Abstract

**Objectives:**

This study aimed to evaluate decision regret following robot‐assisted laparoscopic partial nephrectomy (RALPN) for benign small renal masses (SRMs) in patients who did not undergo preoperative renal mass biopsy (RMB) and to assess factors associated with regret and satisfaction with treatment decision‐making.

**Patients and Methods:**

We conducted a mixed methods study combining retrospective clinical data with prospective patient‐reported outcomes at a high‐volume renal cancer centre. Patients who underwent RALPN for indeterminate SRMs ≤4 cm between January 2010 and July 2025 without preoperative RMB and were subsequently found to have benign histology were identified from a prospectively maintained renal cancer database. Participants completed a standardised questionnaire incorporating the validated Decision Regret Scale (DRS). Significant regret was defined as DRS ≥ 25. Decision regret was evaluated in relation to patients' recollection of counselling and perceived quality of preoperative information.

**Results:**

Of 1480 RALPN procedures performed during the study period, 167 patients met the eligibility criteria. Contact was established with 108 patients, of whom 104 agreed to participate and 58 completed the questionnaire. Median age was 66 years, and median tumour size was 30 mm. No Clavien‐Dindo Grade ≥III complications occurred. Most patients recalled being informed that the lesion could be benign (79.3%). Discussion of RMB was recalled by 55.2% of respondents, whereas 29.3% reported no recollection of biopsy discussion. Overall decision regret was low, with a mean DRS of 13.8 (95% confidence interval [CI] 10.3–17.4), although 27.6% demonstrated clinically significant regret (DRS ≥ 25). Despite benign histology, 87.9% believed surgery had been the correct decision and only 29.3% would retrospectively have preferred RMB before surgery. Lower regret scores were observed in patients who recalled detailed RMB discussion and reported higher perceived quality of preoperative counselling.

**Conclusion:**

Most patients undergoing RALPN for benign small renal masses reported low decision regret despite ultimately benign pathology. Although over one‐quarter of patients demonstrated clinically significant regret, the majority remained satisfied with their treatment decision and believed surgery had been the correct choice. Comprehensive counselling regarding diagnostic uncertainty and the potential role of renal mass biopsy may help minimise subsequent regret, reinforcing the importance of shared decision‐making in contemporary small renal mass management.

## 1 INTRODUCTION

Percutaneous renal mass biopsy (RMB) can provide histological diagnosis for radiologically indeterminate renal masses and may reduce unnecessary surgical intervention for benign pathology. Despite advances in biopsy techniques and tissue processing, the routine use of RMB prior to surgical excision remains varied in contemporary practice. According to the European Association of Urology (EAU) Guidelines on renal cell carcinoma, percutaneous renal mass biopsy is recommended in selected clinical settings, including radiologically indeterminate renal masses where histological confirmation may influence management, small renal masses (SRMs) being considered for active surveillance, prior to ablative treatment, and in metastatic disease to guide systemic treatment and surgical strategies. However, routine biopsy is not recommended for all solid renal masses, particularly where imaging is strongly suggestive of malignancy and the biopsy result is unlikely to alter the planned management.^1^ Similarly, recent NICE guidance recommends consideration of RMB for renal lesions ≤4 cm where histological confirmation may influence management.^2^ Although RMB demonstrates high diagnostic accuracy in experienced centres, non‐diagnostic biopsy rates remain a recognised limitation.^1^, ^3^

SRMs, typically defined as renal masses ≤4 cm, are increasingly detected through cross‐sectional imaging. Contemporary series report that approximately 20%–30% of surgically excised SRMs are ultimately benign on final histology.^4^, ^5^ Consequently, many patients undergo surgical intervention for lesions later confirmed to be benign.

Robot‐assisted laparoscopic partial nephrectomy (RALPN) provides excellent oncological and functional outcomes for appropriately selected SRMs while preserving renal function.^6^ However, surgery carries recognised risks, including bleeding, urine leak, conversion to radical nephrectomy and decline in renal function.^1^, ^7^ Decision‐making therefore requires balancing the risks of intervention against the uncertainty of untreated malignancy.

Decision regret, defined as distress or remorse following a healthcare decision, is an increasingly recognised patient‐reported outcome measure in oncological surgery. In urological oncology, clinically significant regret has been reported in 15%–20% of patients following definitive treatment.^8^, ^9^ Factors associated with regret include treatment related morbidity, unmet expectations, functional outcomes and the quality of shared decision‐making and preoperative counselling.^10^ Although decision regret has been widely studied in prostate cancer, there remains limited evidence evaluating regret following partial nephrectomy for presumed renal cell carcinoma, particularly in patients subsequently found to have benign pathology.

Thus, we aimed to assess the prevalence and determinants of decision regret in this cohort, while also evaluating patient perspectives on preoperative counselling, shared decision‐making and the role of RMB discussion in treatment decision‐making.

## 2 PATIENTS AND METHODS

### 2.1 Study design and patient selection

We conducted a convergent mixed methods study combining retrospective clinical data with prospective patient‐reported outcomes at Frimley Park Hospital, a well‐established specialist centre for kidney cancer with a high‐volume robotic renal surgery practice. Quantitative data were collected through a structured questionnaire incorporating the validated Decision Regret Scale (DRS), while qualitative data were obtained from free‐text patient responses exploring experiences of counselling, decision‐making and treatment. Quantitative and qualitative datasets were analysed separately and subsequently integrated during interpretation to provide a comprehensive assessment of patient perspectives following partial nephrectomy for SRMs.

Patients were identified from a prospectively maintained institutional renal cancer database at Frimley Park. The study was formally registered with the Quality and Audit Team at Frimley Health NHS Foundation Trust. As a registered audit/service evaluation project, formal Research Ethics Committee approval was not required.

Patients who were planned for a nephrectomy for presumed SRM between January 2010 and July 2025 were screened for eligibility.

Inclusion criteria were renal masses ≤4 cm on final histopathology, no preoperative RMB, benign final histology and a minimum follow‐up of 4 months. Patients with solitary kidneys and hilar tumours were included. All patients were planned for and underwent RALPN. No patients initially planned for RALPN were converted to radical nephrectomy.

The exclusion criteria comprised previous renal malignancy, prior RMB, Bosniak cysts, renal masses >4 cm, benign indications for surgery (including radiologically diagnosed or suspected angiomyolipoma [AML], stone disease or nonfunctioning renal moieties), incomplete clinical data, inability to provide informed responses and patients deemed unsuitable for RMB following multidisciplinary team (MDT) discussion. Patients who had died since surgery were also excluded.

Eligible patients were contacted as part of routine follow‐up and invited to complete a standardised questionnaire either electronically, by post or via telephone consultation. A follow‐up telephone call was performed for nonresponders to maximise response rates.

### 2.2 Data collection

Clinical data were retrospectively extracted from electronic patient records (EPIC) and the institutional renal cancer database by two independent investigators (SN and AA), with discrepancies resolved by consensus. Collected variables included patient demographics, tumour characteristics, operative details, histopathological findings and post‐operative outcomes. Post‐operative complications were classified according to the Clavien‐Dindo grading system.

The questionnaire (Appendix S1) incorporated the validated five‐item DRS, together with additional items assessing patient perspectives regarding preoperative counselling, shared decision‐making and discussion of RMB. Open‐ended questions were included to explore patient experiences qualitatively and provide context to the quantitative findings.

### 2.3 Outcomes

The primary outcome was decision regret following RALPN for benign pathology, assessed using the DRS. Scores range from 0 to 100, with higher scores representing greater regret. Clinically significant regret was defined as a DRS ≥ 25.^11^ Secondary outcomes included satisfaction with preoperative counselling, perceptions of shared decision‐making, attitudes towards RMB discussion and the relationship between post‐operative morbidity and regret.

### 2.4 Statistical analysis

Continuous variables were reported as mean (standard deviation [SD]) or median (interquartile range [IQR]) according to data distribution, while categorical variables were summarised as frequencies and percentages. DRS scores were analysed both as continuous variables and categorically according to the presence of clinically significant regret (DRS ≥ 25). Associations between clinicopathological variables and decision regret were assessed using Student's *t*‐test, Mann–Whitney *U* test, chi‐square test or Fisher's exact test, as appropriate. Statistical analyses were performed using IBM SPSS Statistics version 28.0 (IBM Corp., Armonk, NY, USA).

Free‐text responses underwent inductive thematic analysis following the six‐phase approach described by Braun and Clarke.^12^ Two investigators (SN and AA) independently familiarised themselves with the data, generated initial codes and collated these into candidate themes, which were subsequently reviewed, defined and named through discussion until consensus was reached.

## 3 RESULTS

### 3.1 Study population

Fifty‐eight patients completed the questionnaire (response rate 55.8% from 104 participants) (Figure 1). The cohort was predominantly White British/Irish/Other (94.8%), with mixed educational backgrounds: 36.2% held a university degree or higher, while 56.9% had A‐level education or below. Median age was 66 years, and the median tumour size was 30 mm. All procedures were performed robotically. No Clavien‐Dindo Grade III or higher complications were recorded. The histological subtypes of the benign lesions are summarised in Table 1.

**FIGURE 1:**
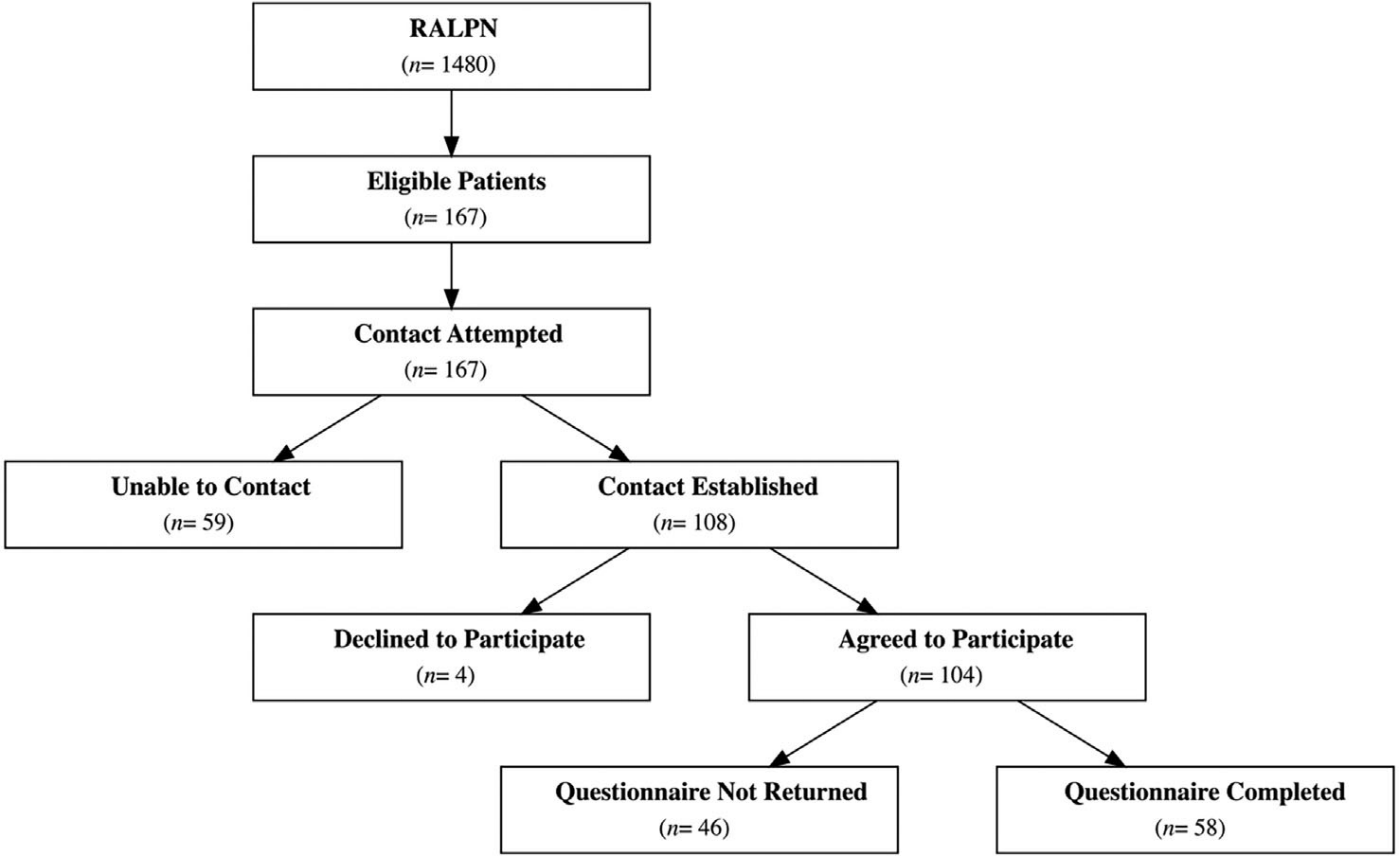
CONSORT diagram on questionnaire responses by patients.

**TABLE 1:** Cohort characteristics (*n* = 58).

Patient characteristics	
Demographics	
Sex, *n* (%)	
Male	30 (51.7)
Female	28 (48.3)
Ethnicity, *n* (%)	
White British/Irish/Other	55 (94.8)
Asian/Asian British	2 (3.4)
Black/African/Caribbean/Black British	1 (1.7)
Education, *n* (%)	
Secondary/GCSE	18 (31.0)
College/A‐levels	15 (25.9)
University degree	10 (17.2)
Postgraduate degree	11 (19.0)
Other qualification	4 (6.9)
Clinical and operative characteristics	
Median age, years	66
Median tumour size, mm	30
Clavien‐Dindo Grade ≥III complications, *n* (%)	0
Histology, *n* (%)	
Oncocytoma	52 (89.7)
Angiomyolipoma (AML)	4 (6.9)
Simple cortical cyst	1 (1.7)
Haemangioma	1 (1.7)

### 3.2 Preoperative counselling

The majority of patients (79.3%) recalled being informed preoperatively that their renal mass could potentially be benign (Table 2). Biopsy was discussed with 55.2% of patients, though 34.5% recalled detailed discussion. Satisfaction with preoperative information was high, with 98.3% rating the quality as excellent or good.

**TABLE 2:** Patient counselling and treatment decision‐making (*n* = 58).

Patient counselling	
Preoperative counselling	
Informed mass could be benign, *n* (%)	
Yes	46 (79.3)
Unsure	7 (12.1)
No	5 (8.6)
Renal mass biopsy discussed, *n* (%)	
Yes, in detail	20 (34.5)
Yes, but minimally	12 (20.7)
No	17 (29.3)
Cannot recall	9 (15.5)
Quality of information, *n* (%)	
Excellent	34 (58.6)
Good	23 (39.7)
Poor	1 (1.7)
Treatment Decision‐Making	
Reasons for surgery, *n* (%)^a^	
Following surgeon's recommendation	35 (60.3)
Remove potential cancer	28 (48.3)
Peace of mind	18 (31.0)
Concern about mass enlarging	16 (27.6)
Previous experience of cancer	7 (12.1)
Decision involvement, *n* (%)^a^	
Shared with surgeon/medical team	29 (50.0)
With partner/spouse	14 (24.1)
Alone	11 (19.0)
With other family	11 (19.0)
Mainly clinician's decision	3 (5.2)
Reasons for declining biopsy, *n* (%)^a^	
Preference to remove regardless	12 (20.7)
Concern about complications	4 (6.9)
Surgeon recommended against	4 (6.9)
Concern about diagnostic accuracy	2 (3.4)
Not applicable/not discussed	17 (29.3)

^a^ Categories not mutually exclusive (respondents could select more than one option).

### 3.3 Treatment decision‐making

The most frequently cited reasons for choosing surgery were following the surgeon's recommendation (*n* = 35, 60.3%) and a wish to remove a potential cancer (*n* = 28, 48.3%), followed by peace of mind (*n* = 18, 31.0%), concern about the mass enlarging (*n* = 16, 27.6%) and previous personal or family experience of cancer (*n* = 7, 12.1%) (Table 2). Half of respondents (*n* = 29, 50.0%) described the decision as shared with their surgeon or wider medical team, while 24.1% (*n* = 14) involved a partner or spouse and 19.0% (*n* = 11) reported deciding alone; only 5.2% (*n* = 3) felt the decision had been made principally by the clinician. Among patients for whom biopsy was discussed, the most common reasons for not proceeding with it were a preference to remove the mass irrespective of the biopsy result (*n* = 12, 20.7%), concern about biopsy‐related complications (*n* = 4, 6.9%) and a surgeon recommendation against biopsy (n = 4, 6.9%), with concern about accuracy cited less often (*n* = 2, 3.4%).

### 3.4 Decision regret

Overall decision regret was low. The mean DRS score was 13.8 out of 100 (SD 13.5), with a median of 11.2 (IQR 0.0–25.0). Using the established threshold of DRS ≥ 25 for clinically significant regret, 72.4% of patients (42/58) had no significant regret. Only 5.2% of the cohort scored in the high regret category (DRS ≥ 50) (Table 3).

**TABLE 3:** Decision reflection (*n* = 58).

Decision reflection	
Decision Regret Scale (DRS)	
Mean (SD)	13.8 (13.5)
Median (IQR)	11.2 (0.0–25.0)
Range	0–55
DRS category, *n* (%)	
No regret (0)	16 (27.6)
Low (1–24)	26 (44.8)
Moderate (25–49)	13 (22.4)
High (≥50)	3 (5.2)
No significant regret (DRS < 25)	42 (72.4)
Significant regret (DRS ≥ 25)	16 (27.6)
Individual DRS items, *n* (%) Agree/Strongly Agree	
Satisfied with decision	58 (100)
Decision was right for me	57 (98.3)
Would make same choice again	52 (89.7)
Regret my choice	3 (5.2)
Choice did me harm	2 (3.4)
Patient‐reported reflections, *n* (%) Agree/Strongly Agree	
Surgery still right despite benign histology	51 (87.9)
Would have preferred biopsy first	17 (29.3)
[Disagree/strongly disagree]	29 (50.0)

*Note*: 0–100, higher = greater regret; significant regret defined as DRS ≥ 25.

Abbreviations: DRS, decision regret scale; IQR, interquartile range; SD, standard deviation.

^a^ Categories not mutually exclusive (respondents could select more than one option).

Examination of the individual scale items reflected this overall pattern (Figure 2). All respondents agreed or strongly agreed that they were satisfied with their decision (*n* = 58, 100%), that the decision had been the right one for them (*n* = 57, 98.3%) and that they would make the same choice again (*n* = 52, 89.7%). Conversely, only 5.2% (*n* = 3) agreed that they regretted their choice and only 3.4% (*n* = 2) felt the decision had caused them harm. When categorised, 27.6% (*n* = 16) of patients recorded a score of 0, indicating no regret.

**FIGURE 2:**
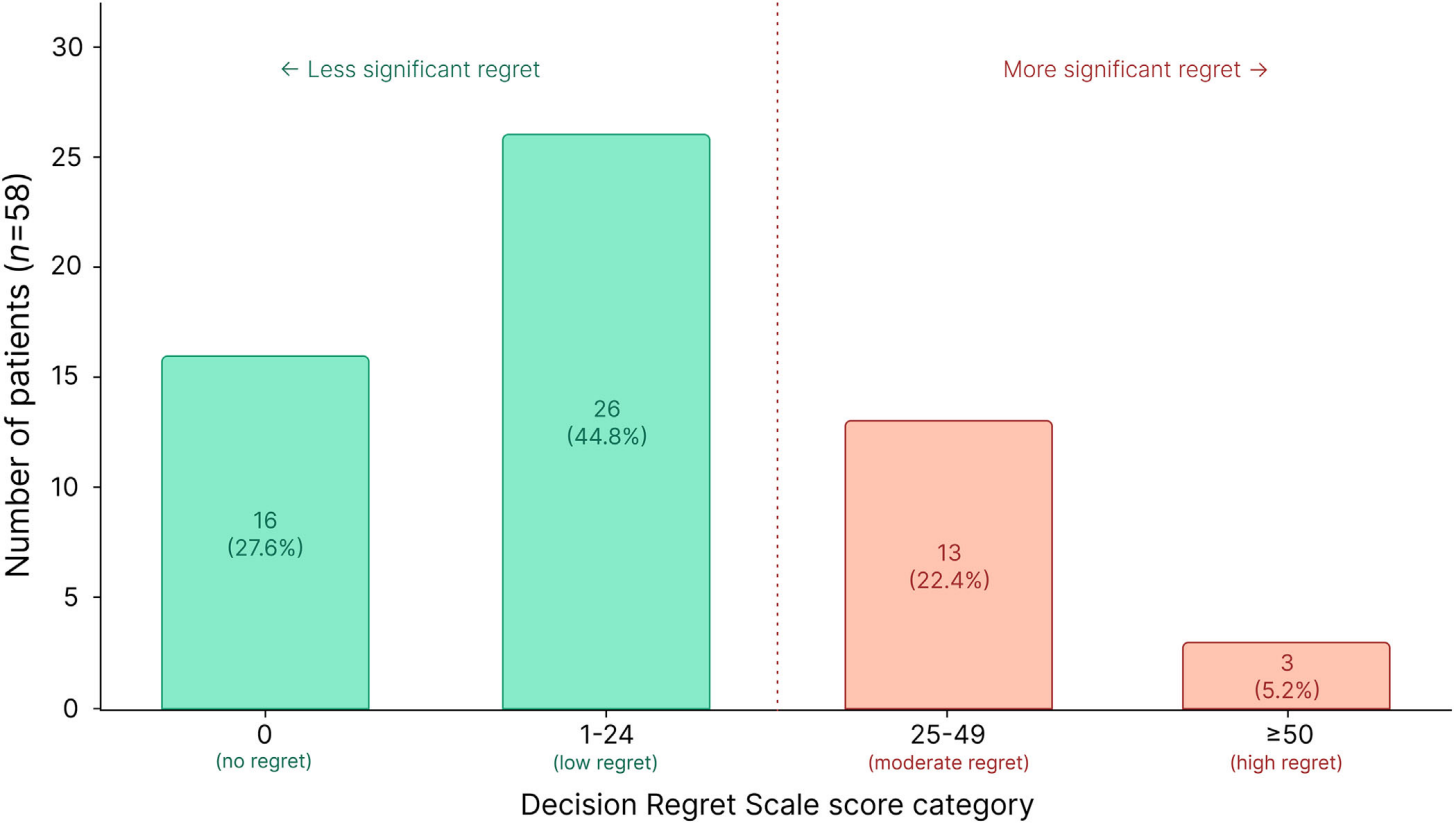
DRS distribution.

When analysed by counselling quality, patients who recalled detailed biopsy discussion had substantially lower regret (mean DRS 8.5) compared to those with no discussion (mean DRS 17.1) (Figure 3). Similarly, patients rating information quality as excellent had lower regret (mean DRS 9.7) than those rating it as good (mean DRS 19.5).

**FIGURE 3:**
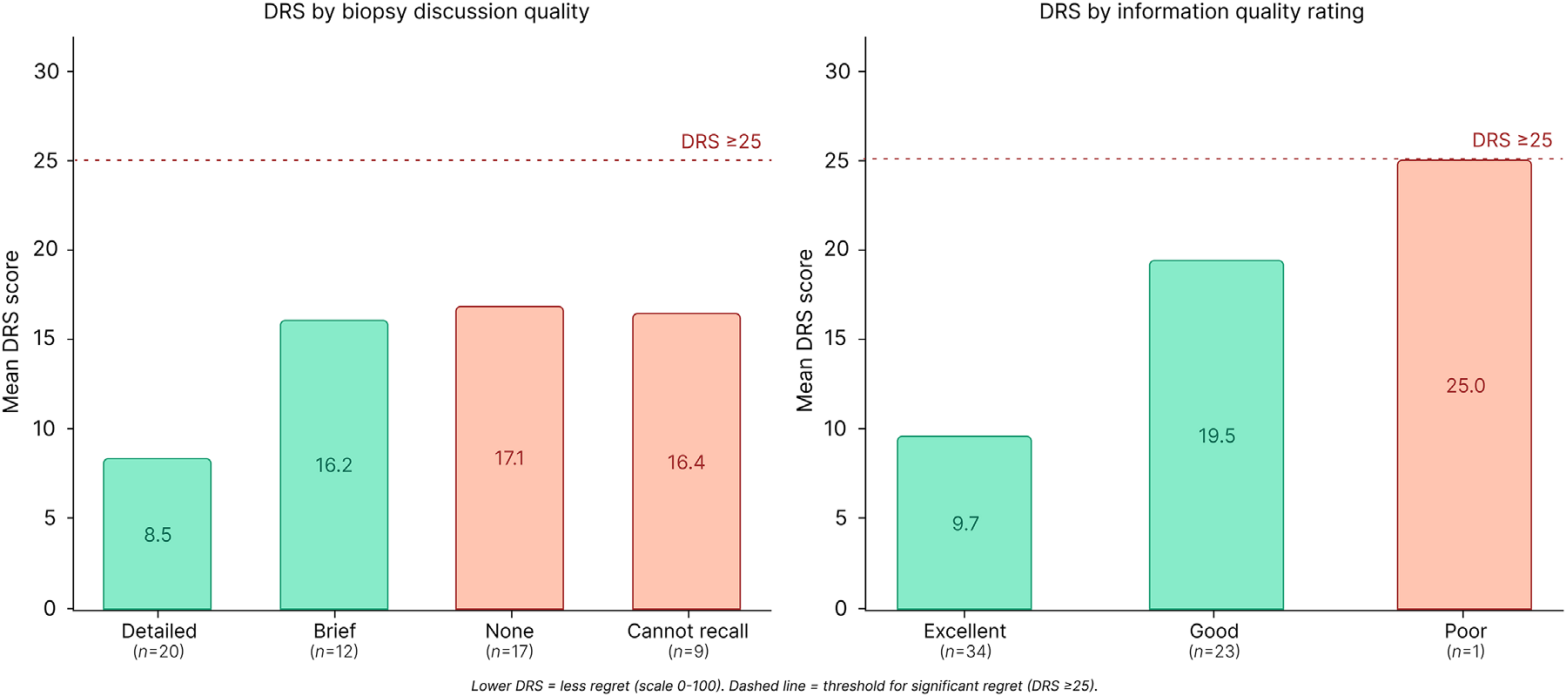
Decision regret scores according to quality of renal mass biopsy discussion and perceived quality of preoperative information.

### 3.5 Patient reflections

Two further questions explored patients' reflections in light of the subsequently confirmed benign histology. The majority, 87.9% (*n* = 51), still believed surgery had been the right decision despite the benign result, with only one patient (1.7%) disagreeing. Retrospective views on biopsy were more divided: 29.3% (*n* = 17) would have preferred a biopsy first, half (*n* = 29, 50.0%) actively disagreed with wanting a biopsy beforehand, and the remainder were neutral.

### 3.6 Qualitative findings

Free‐text comments were provided by the majority of respondents (*n* = 56) (Table 4). Responses were collected through three open questions and grouped into three principal themes: advice to future patients (*n* = 55), gaps in preoperative information (*n* = 16) and reflections on the overall care experience (*n* = 45). As respondents could contribute to more than one question, these categories are not mutually exclusive.

**TABLE 4:** Thematic analysis of free‐text responses (*n* = 56 respondents).

Theme	Subthemes (*n*)	Illustrative quotations
Advice to future patients (*n* = 55)	Proceed with surgery (*n* = 24); trust the clinical team (*n* = 14); seek biopsy where feasible (*n* = 10); individual deliberation (*n* = 9); seek information/second opinion (*n* = 8)	*‘Definitely have the surgery done'; 'Trust what the specialists say'; 'If possible opt for biopsy because it might avoid surgery’*
Gaps in preoperative information (*n* = 16)	Post‐operative pain/recovery (*n* = 5); benign possibility or biopsy option (*n* = 7); institutional outcome data (*n* = 2)	*‘It was extremely painful afterwards. That wasn't explained’; ‘Always thought [they] would have taken a biopsy first but they didn’t*
Reflections on care experience (*n* = 45)	Positive care/staff (*n* = 26); communication of results/follow‐up (*n* = 14); in‐patient experience during service pressures (*n* = 12); post‐operative pain/side‐effects (*n* = 7)	*‘The specialist nurse was wonderful and very helpful’; ‘(The surgeon) and his team were absolutely superb’*

*Note*: Themes derived from three open‐ended questions using inductive thematic analysis (Braun and Clarke).^12^ As respondents could answer more than one question, themes are not mutually exclusive.

Advice to prospective patients most commonly encouraged proceeding with surgery (*n* = 24), frequently expressed in direct terms such as, ‘Definitely have the surgery done’ and ‘Get it out, have the surgery’. A second strand emphasised trust in the clinical team (*n* = 14), for example, ‘Trust what the specialists say’ and ‘Listen to the professionals, take their advice’, while a smaller group advocated individual deliberation (*n* = 9), including: ‘Make up your own mind’ and ‘Do what feels right for yourself’. A minority specifically advised seeking biopsy where feasible (*n* = 10), such as, ‘Have a biopsy first’ and ‘If possible opt for biopsy because it might avoid surgery’.

Although most patients reported having felt well‐informed, those identifying a specific information gap (*n* = 16) most often cited the severity and duration of post‐operative pain and recovery (*n* = 5), for example, ‘It was extremely painful afterwards. That wasn't explained’, together with a wish for clearer discussion of the possibility of benign disease and of the biopsy option (*n* = 7), such as, ‘Always thought [they] would have taken a biopsy first but they didn't’. A small number requested institutional outcome data to inform their choice (*n* = 2).

Reflections on the care experience (*n* = 45) were predominantly positive (*n* = 26), with frequent praise for the surgical and nursing teams, such as, ‘The specialist nurse was wonderful and very helpful’ and ‘(The surgeon) and his team were absolutely superb’. Where concerns were raised, these related chiefly to post‐operative pain or medication side‐effects (*n* = 7), the in‐patient experience during periods of service pressure (*n* = 12) and the communication of results, with several patients expressing a preference for written confirmation of histology rather than by telephone alone (*n* = 14).

## 4 DISCUSSION

The increasing detection of SRMs through cross‐sectional imaging has led to a growing clinical challenge regarding overtreatment of indeterminate renal lesions. The principal finding of this study is that most patients remained satisfied with their decision to undergo surgery despite ultimately benign pathology, although over one‐quarter experienced clinically significant regret. Importantly, patients who recalled more detailed counselling regarding diagnostic uncertainty and renal mass biopsy reported lower regret scores, highlighting the importance of high‐quality shared decision‐making in small renal mass management. The mean DRS score in our cohort was 13.8, which is lower than regret rates reported across several other urological oncological settings, including prostate cancer treatment.^8^, ^13^ Furthermore, only 5.2% of patients demonstrated high regret (DRS ≥ 50). These findings are important as they suggest that patients may derive reassurance and psychological benefit from definitive surgical management despite ultimately benign histology. Qualitative responses additionally suggested that anxiety regarding potential cancer progression and the desire for diagnostic certainty were major contributors influencing treatment choice.

Our findings differ substantially from those reported by Mendhiratta et al.,^14^ who surveyed patients with benign renal masses and found clinically significant decisional regret in 49% of respondents, with a mean DRS score of 32. In contrast, the mean DRS score in our cohort was 13.8 and only 27.6% of patients demonstrated clinically significant regret. Importantly, Mendhiratta et al. identified a lack of preoperative discussion regarding the possibility of benign pathology and available management options as a common source of regret, with qualitative responses highlighting unmet expectations and insufficient counselling. Similar themes emerged in our study; however, most patients recalled being informed that their lesion could be benign and reported high satisfaction with the quality of information provided. Furthermore, patients who recalled detailed discussion regarding renal mass biopsy demonstrated lower regret scores. Taken together, these findings suggest that while benign pathology following surgery may be associated with decisional regret, comprehensive counselling regarding diagnostic uncertainty and management options may mitigate this effect and improve patient satisfaction.

The pattern of lower regret among patients reporting more detailed counselling in both studies further supports the importance of structured shared decision‐making and informed discussions regarding diagnostic uncertainty when managing indeterminate SRM. Patients who recalled detailed discussion regarding RMB demonstrated substantially lower regret scores compared with those reporting minimal or absent biopsy discussion. Similarly, patients reporting higher perceived quality of preoperative information had lower decision regret scores. This aligns with broader oncological literature demonstrating that unmet expectations and inadequate involvement in shared decision‐making are strongly associated with subsequent decision regret.^9^, ^15^ Importantly, our findings do not necessarily support universal utilisation of RMB prior to surgery. Although RMB can improve diagnostic certainty and potentially avoid surgery for benign lesions, its role should be individualised. Limitations include non‐diagnostic sampling and the potential for sampling error, particularly in heterogeneous lesions, while recognised complications include infection, bleeding and perinephric haematoma, although clinically significant complications are uncommon. These limitations should form part of shared decision‐making when RMB is considered alongside surveillance or definitive treatment. Emerging noninvasive imaging modalities, including ^99m^Tc‐sestamibi SPECT/CT^16^ and ^89^Zr‐girentuximab PET‐CT, may provide additional information regarding malignancy risk and further support treatment selection in appropriately selected patients.^17^, ^18^

Interestingly, despite ultimately benign pathology, fewer than one third of patients retrospectively wished they had undergone RMB before surgery. This likely reflects the complex psychological context surrounding SRM management, where fear of malignancy frequently influences patient decision‐making. Similar findings have been observed in studies evaluating patient preferences in active surveillance and renal mass management, where many patients favour definitive treatment despite relatively low oncological risk.^19^, ^20^ Notably, Patel et al.^21^ observed that patients who underwent surgical intervention reported a better physical quality of life, albeit a similar mental quality of life, compared to those managed with active surveillance, and these scores improved over time in both groups. These observations highlight that treatment decisions in SRMs are influenced not only by oncological outcomes but also by psychological reassurance, risk perception and individual tolerance of uncertainty.

The present study has several important clinical implications. First, the findings reinforce the importance of structured shared decision‐making when counselling patients with indeterminate SRMs. Second, the study highlights that discussion of RMB itself may hold value beyond simply obtaining tissue diagnosis, serving as part of a broader patient‐centred counselling process. Finally, these findings may help clinicians better contextualise discussions regarding overtreatment, active surveillance and biopsy utilisation in contemporary SRM practice.

Several limitations should be acknowledged. This was a single centre study performed within a high‐volume robotic renal surgery unit, which may limit generalisability. The modest sample size and response rate introduce potential responder bias and patient recollection of counselling may be affected by recall bias given the retrospective nature of the questionnaire assessment. The median interval between surgery and questionnaire completion was 61 months (range 4–184 months), meaning that some participants were asked to recall consultations and treatment decisions several years after treatment, potentially influencing the accuracy of reported counselling experiences and perceptions of shared decision‐making.

In addition, the absence of a comparator cohort undergoing preoperative RMB limits conclusions regarding the direct impact of biopsy on decision satisfaction. Finally, the cohort experienced low rates of major post‐operative morbidity, which may partly explain the overall low regret scores observed.

Nevertheless, to our knowledge, this is the first study specifically evaluating decision regret following partial nephrectomy for presumed malignant SRMs subsequently found to have benign pathology in patients who had not undergone preoperative RMB. Most patients who underwent partial nephrectomy for presumed malignant small renal masses subsequently found to be benign reported low levels of decision regret and remained satisfied with their treatment decision. However, more than one‐quarter experienced clinically significant regret. Detailed discussion of diagnostic uncertainty and the potential role of renal mass biopsy was observed in patients with lower decision regret, suggesting that the quality of preoperative counselling may be an important modifiable factor influencing patient experience. Hence, further studies are needed to assess in depth the quality of patient counselling and how to improve this to minimise decision regret. These findings support structured shared decision‐making and comprehensive counselling as integral components of contemporary small renal mass management.

## AUTHOR CONTRIBUTIONS

SN, AA and NB conceived the study, developed the methodology, and designed the questionnaire. MY and SD contributed to questionnaire distribution and data collection. SN, AA and BG led the data interpretation, manuscript drafting, and preparation. All authors contributed to the review and critical revision of the manuscript and approved the final version for publication.

## CONFLICT OF INTEREST STATEMENT

Aqua Asif is funded via the NIHR (Academic Clinical Fellowship). No other conflicts of interest exist.

## Supporting information


**Data S1.** Supporting Information

## Data Availability

The data that support the findings of this study are available from the corresponding author upon reasonable request.
