# Supplementary material for: Decision regret after partial nephrectomy for benign small renal masses: The importance of preoperative counselling
Source: BJUI Compass. 2026 Oct 16;7(10):e70289. doi: 10.1002/bco2.70289 (PMC13673402; doi:10.1002/bco2.70289)
Supplement: Supplementary file 1 [file BCO2-7-e70289-s001.docx]

# Supplementary Appendix 1

## Questionnaire

### Section 1: Demographics

1. Ethnicity:
   - White - British/Irish/Other
   - Black/African/Caribbean/Black British
   - Asian/Asian British
   - Mixed/Multiple ethnic groups
   - Other (please specify): _________________
   - Prefer not to say
2. Highest level of education completed:
   - Primary school
   - Secondary school/GCSE
   - College/A-levels
   - University degree
   - Postgraduate degree
   - Other (please specify): _________________
   - Prefer not to say

### Section 2: Pre-Operative Experience

1. Before your surgery, do you recall being informed that your kidney mass could potentially be benign (non-cancerous)?
   - Yes
   - No
   - Unsure
2. Was the option of a kidney biopsy (to determine if the mass was cancerous) discussed with you before your operation?
   - Yes, in detail
   - Yes, but minimally
   - No
   - Cannot recall
3. If a biopsy was discussed, what were your main reasons for not having one? (Select all that apply)
   - Concern about biopsy complications
   - Concern about biopsy diagnostic accuracy
   - Preference to remove the mass regardless of biopsy results
   - Surgeon recommended against biopsy
   - Not applicable - biopsy was not discussed
   - Other (please specify): _________________
4. What were your main reasons for choosing to have partial nephrectomy surgery? (Select all that apply)
   - Remove potential cancer
   - Peace of mind
   - Following surgeon's recommendation
   - Previous experience with cancer (self or family/friends)
   - Concerns about mass growing larger
   - Other (please specify): _________________
5. Who was involved in making the decision about your treatment? (Select all that apply)
   - Myself alone
   - Myself and my partner/spouse
   - Myself and other family members
   - Myself and my surgeon/doctor/medical team
   - Mainly the surgeon/doctor's decision
   - Other (please specify): _________________
6. How would you rate the quality of information you received before deciding on surgery?
   - Excellent - I felt fully informed
   - Good - I felt adequately informed
   - Fair - I had some information but wanted more
   - Poor - I felt inadequately informed
   - Very poor - I felt uninformed

### Section 3: Decision Satisfaction

For the following statements, please indicate your level of agreement (select one option for each statement):

1. I am satisfied with the decision I made to have partial nephrectomy surgery
   - Strongly agree
   - Agree
   - Neither agree nor disagree
   - Disagree
   - Strongly disagree
2. The decision to have surgery was the right one for me
   - Strongly agree
   - Agree
   - Neither agree nor disagree
   - Disagree
   - Strongly disagree
3. If I had to do it again, I would make the same choice
   - Strongly agree
   - Agree
   - Neither agree nor disagree
   - Disagree
   - Strongly disagree
4. I regret the choice that I made
   - Strongly agree
   - Agree
   - Neither agree nor disagree
   - Disagree
   - Strongly disagree
5. The choice I made did me a lot of harm
   - Strongly agree
   - Agree
   - Neither agree nor disagree
   - Disagree
   - Strongly disagree
6. Now that I know my mass was benign (non-cancerous), I still believe having surgery was the right decision
   - Strongly agree
   - Agree
   - Neither agree nor disagree
   - Disagree
   - Strongly disagree
7. Knowing what I know now, I would have preferred to have had a biopsy first
   - Strongly agree
   - Agree
   - Neither agree nor disagree
   - Disagree
   - Strongly disagree

### Section 4: Additional Perspectives

1. What information do you wish you had known before your surgery?
2. What advice would you give to someone in a similar situation?
3. Is there anything else you would like to share about your experience?
